# Venetoclax plus cyclophosphamide and topotecan in heavily pre-treated relapsed metastatic neuroblastoma: a single center case series

**DOI:** 10.1038/s41598-023-44993-9

**Published:** 2023-11-07

**Authors:** Maria Antonietta De Ioris, Francesco Fabozzi, Francesca Del Bufalo, Giada Del Baldo, Maria Felicia Villani, Maria Giuseppina Cefalo, Maria Carmen Garganese, Alessandra Stracuzzi, Federica Tangari, Arturo Maria Greco, Isabella Giovannoni, Roberto Carta, Maria Luisa D’Andrea, Angela Mastronuzzi, Franco Locatelli

**Affiliations:** 1 Department of Pediatric Hematology and Oncology and of Cell and Gene Therapy, Bambino Gesù Children’s Hospital, IRCCS, Rome, Italy; 2 Nuclear Medicine Unit, Bambino Gesù Children’s Hospital, IRCCS, Rome, Italy; 3 Pathology Unit, Bambino Gesù Children’s Hospital, IRCCS, Rome, Italy; 4 Unit of Clinical Pharmacy, Bambino Gesù Children’s Hospital, IRCCS, Rome, Italy; 5 Department of Imaging, Bambino Gesù Children’s Hospital, IRCCS, Rome, Italy; 6 Department of Life Sciences and Public Health, Catholic University of the Sacred Heart, Milan, Italy

**Keywords:** Cancer therapy, Paediatric cancer

## Abstract

The prognosis of relapsed/refractory (R/R) neuroblastoma (NB) is dismal, calling for new therapeutic strategies. Venetoclax (VEN) is a highly selective, potent, orally bioavailable, BCL-2 inhibitor small-molecule that showed a synergistic effect with cyclophosphamide and topotecan (Cy-Topo) in murine NB models. Our aim was to evaluate the feasibility of VEN plus Cy-Topo in children with R/R NB. Four patients, who had previously failed > 3 lines of treatment, were treated with VEN plus Cy-Topo based on a 28-day schedule in an outpatient setting. BCL-2 expression in immunochemistry on tumor samples at relapse and the *BCL2* gene status was evaluated in all patients. The main toxicity was hematological, with grade 4 neutropenia and thrombocytopenia occurring in all courses and leading to transient VEN discontinuation. Grade 3 oral mucositis was observed in 1/8 courses. No other grade 2–4 toxicities were observed. BCL-2 was expressed in all tumors, while no molecular abnormalities in the *BCL-2* genes were detected. A stable disease was observed in all patients, without any progression during the study period. VEN plus Cy-Topo is well tolerated, with encouraging results that may be improved by testing the schedule in less advanced patients.

## Introduction

Neuroblastoma (NB) represents the most frequent extracranial solid tumor of childhood. The prognosis ranges from a spontaneously tumor regression to a poor outcome despite a multimodal treatment^1,2^. Patients with high-risk (HR) NB present a refractory/relapsed (RR) disease in about 50% of cases, and the prognosis of these patients is grim with an Event-Free Survival (EFS) < 10%, calling for new therapeutic strategies^3,4^. The BCL-2 protein family controls the commitment to programmed cell death by preventing activation of the mitochondrial apoptosis pathway, resulting in enhanced cell survival^5^. The BCL-2 family proteins are often found overexpressed in NB cells, thus representing a promising target for new approaches in the treatment of NB^6–8^. Venetoclax (VEN) is a highly selective, potent, orally bioavailable small-molecule inhibitor of the BCL-2 proteins, leading to a restoration of apoptosis^9^. It was found both safe and effective in children affected by hematological malignancies, including acute myeloid leukemia, acute lymphoblastic leukemia and lymphoblastic lymphoma^10–12^. The studies in NB murine xenografts showed a modest activity as single agent, while VEN has a synergistic effect when combined with cyclophosphamide and topotecan (Cy-Topo)^7,13^. Its use in the clinic setting is currently under investigation (NCT03236857)^14^. Early results showed that one patient with NB achieved complete remission (CR), whereas in most cases continuous dosing of VEN with Cy-Topo was not tolerated due to cytopenia^15^.

Herein, we report a real-life case series of four heavily pre-treated patients affected by R/R NB treated with VEN + Cy-Topo at our institution. In all patients, we evaluated BCL-2 expression on tumor tissue. The 4 children received a 400-mg adult exposure-equivalent target dose, in keeping with NCT03236857 trial^14^.

## Cases description

Between April 2022 and October 2022, 4 patients with heavily pretreated metastatic R/R NB were treated with two courses of Cy-Topo plus VEN. The proposed course was based on a 28-day schedule: on day 1 of week 1, VEN was started at 200-mg adult-equivalent dose, increased to 400-mg adult-equivalent dose on day 2, if tolerated, and was then administered daily throughout the entire cycle. In week 2, Cy was administered at the dose of 250 mg/m^2^/day for 5 days, together with Topo, at the dose of 0.75 mg/m^2^/day for 5 days. VEN was discontinued only in case of severe neutropenia (absolute neutrophil count, ANC, < 500/mmc) and re-started upon resolution. Hematologic criteria to initiate a subsequent cycle were ANC > 500/mmc and platelet count > 50,000/mmc.

The Internal Review Board (IRB) of the Bambino Gesù Children’s Hospital approved the administration of the proposed schedule in each patient and a specific informed consent was signed by the parents or legal guardians. All investigations were conducted in accordance with the principles expressed in the Declaration of Helsinki.

Overall, four patients were treated with the proposed schedule. All these patients presented a R/R NB and previously were given more than three lines of treatment. Specifically, all patients received standard treatment for HR NB, multiple courses of chemotherapy after relapse, including previous exposure to Cy and Topo, high dose chemotherapy, and immunotherapy with dinutuximab beta; moreover, radiometabolic therapy with 131I-metaiodobenzylguanidine (MIBG) was administered to 3 out of 4 and experimental immunotherapy with chimeric antigen receptor (CAR)-T cells targeting the disialoganglioside-2 (GD2) was administered in two out of four patients^16^. Patients’ characteristics including the disease status and the response according to the International Neuroblastoma Response Criteria (INRC) and SIOPEN skeletal score^17,18^ after 2 VEN-Cy-Topo courses are summarized in Table 1, while toxicities according to the Common Terminology Criteria for Adverse-Events (CTCAE v5.0) are detailed in Table 2. The main toxicity was hematological: grade 4 neutropenia occurred in all courses, leading to temporary VEN discontinuation in all patients, as well as grade 4 thrombocytopenia. However, all patients received VEN for at least two weeks consecutively. Febrile neutropenia complicated two out of 8 courses. Of note, it was necessary to delay planned administration of Cy-Topo only in one patient who experienced febrile neutropenia; treatment was restarted with a 4-week delay, at the reduced dose of 30%. Grade 3 oral mucositis was recorded during one of 8 courses, whereas no other extra hematological grade 2–4 toxicities were observed.

**Table 1:** Disease status and response after 2 VEN plus Cy-Topo courses.

Pt	Sex	Age (mo)	Disease status before treatment	MYCN status	Previous Therapy	BCL2 IHC	Score 0	Score 1	Response INRC	TTE	Further treatments	Follow-up time (mo)	Last follow-up
1	F	65	Bone/Bone-Marrow Soft Tissue (abdomen paravertebral, sternum)	gain	4	80%, diffuse	8	5	SD	5	yes	18	alive, SD
2	M	81	Bone/Bone-Marrow Soft Tissue (Skull plus soft tissue)	gain	3	80%, diffuse	6	5	SD	9	no	9	alive, PD
3	M	101	Bone/Bone-Marrow	NA	5	10%, focal	5	5	SD	2.5	yes	14	alive, PD
4	M	82	Bone/Bone-Marrow	NA	5	10%, focal	25	22	SD	2	yes	12	alive, PD

Pt, patients; Age, age at treatment; mo, months; Not Amplified, NA; Score 0; MIBG Skeletal Score before VEN plus Cy-Topo; Score 1; MIBG Skeletal Score after two courses of VEN plus Cy-Topo; Previous Therapy, previous treatment lines before VEN plus Cy-Topo; BCL2 IHC, immunoistochemical BCL-2 expression; INRC, International Neuroblastoma Response Criteria. TTE, time-to-event (in all cases the event was another treatment, no PD occurred during the study period).

**Table 2:** Treatment toxicities were resumed according to CTCAE v5.0.

Patients	Anemia (grade)	Max duration (days)	Decreased N count (grade)	Max duration (days)	Decreased PLTs count (grade)	Max duration (days)	Febrile neutropenia (grade)	Others (grade)	VEN withdrawal	Cy-Topo dose reduction	Delayed Cy-Topo
1	3	1	4	29	4	8	No	NA	Yes (2 weeks)	No	No
2	3	1	4	24	4	3	4	oral mucositis (G3)	Yes (4 weeks)	Yes	Yes
3	3	1	4	16	4	10	No	NA	Yes (2 weeks)	No	No
4	3	1	4	5	4	9	3	NA	Yes (3 weeks)	Yes	No

N, neutrophil; PLTs, platelets; VEN, venetoclax; Cy-Topo, cyclophosphamide and topotecan; NA, not applicable.

A stable disease (SD) disease was observed in all patients, without any progression in the study period. In addition, a slight improvement of MIBG SIOPEN skeleton score was observed in 3 out four cases (Supplementary Fig. S1 and Supplementary Table S1).

Of note, patient 2 underwent tumor biopsy with the aim of searching for possible additional molecular targets. Surprisingly, histological examination showed Schwannian stroma-poor NB, with only differentiating or necrotic tissue (Supplementary Fig. S2).

**Figure 1:**
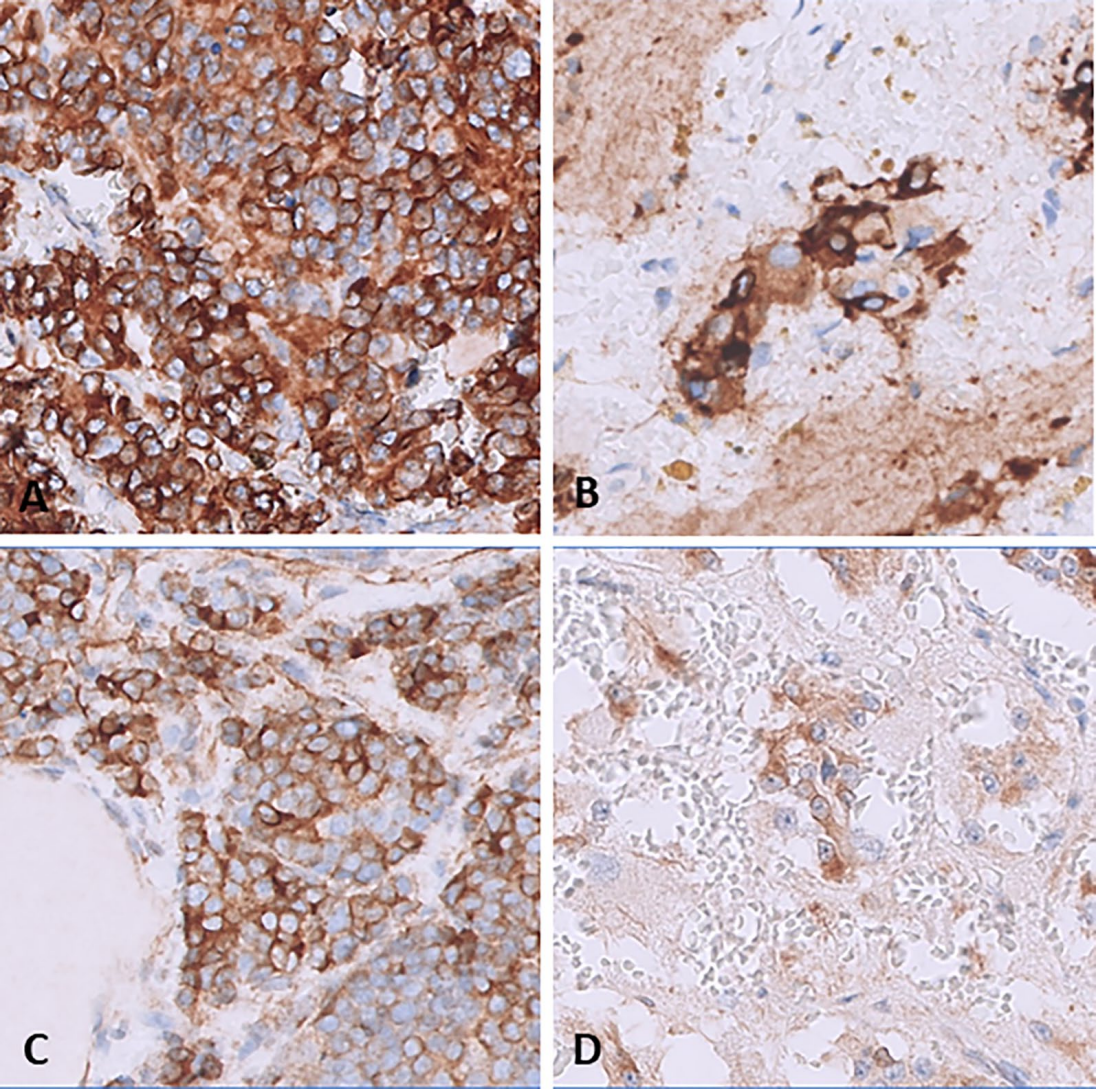
BCL-2 immunostain. Diffuse intense cytoplasmic staining is seen in patient 1 (**A**) and 2 (**B**), while mild to moderate staining is seen in patient 3 (**C**) and 4 (**D**).

Immunohistochemical (IHC) staining for BCL-2 was performed (Mouse, clone 124, Dako, Ready to use) on paraffin-embedded tissue samples from all patients of our series treated with VEN on The DAKO OMNIS platform (Fig. 1). Three patients diagnosed with neuroblastoma (schwannian stroma-poor), poorly differentiated, at onset were used as a comparison group. A semiquantitative scoring system (0, negative; 1, mild; 2, moderate; 3, strong) was applied, with scores incorporating combined prevalence of the intensity of tumor cells labeling and the percentage of positive neoplastic cells. In all 7 patients (4 cases and 3 controls) cytoplasmic staining was detected in a percentage of neoplastic cells ranging from 10 to 80%.

A comprehensive genomic profiling on tumor paraffin-embedded tissue samples was performed in 3 out of 4 patients and no alterations of *BCL-2* gene status was detected.

Therefore, these results may suggest a constitutive expression of BCL-2 in NB tumor cells linked to the activation of the anti-apoptotic gene pathway.

## Discussion

During the last three decades different second-line strategies have been proposed in children with R/R NB. Traditional approaches include salvage chemotherapy with different combinations, such as temozolomide/irinotecan (TEMIRI), Cy-Topo, and Topo/Temozolomide (TOTEM), with similar outcomes^19–21^. A valuable alternative is represented by 131I-MIBG radiometabolic therapy, with refractory disease seeming to fare better than relapsed NB^22^. However, the prognosis of R/R NB remains dismal, especially for patients with HR disease at diagnosis^23^, calling for newer approaches. In this regard, the addition of immunotherapy to chemotherapy seems to improve the effectiveness of standard backbone treatments^24,25^, whereas molecular targeted treatments seem limited in the NB setting. Currently, the most druggable target for neuroblastoma is represented by anaplastic lymphoma kinase (ALK), as activating *ALK* mutations and amplifications are detected in approximately 10% and 4.5% of neuroblastoma tumors, respectively^26^. The effort to face R/R NB is consistent with more than 150 phase 1 and 2 trials from 2011 to 2020 as recently reported^27^.

For NB patients who fail first-line therapies there are very few treatments to offer in order to achieve long-term disease control. A very attractive approach is the use of CAR T cells against GD2, expressed on NB cells^16^ and transplantation of haploidentical stem cells followed by the anti-GD2 antibody dinutuximab beta^28^. In both strategies, a better outcome correlates with low disease burden. In this scenario, strategies to stabilize and/or reduce the disease burden become crucial, but for these highly pretreated patients the available weapons are extremely limited. In this perspective, the combination of chemotherapy and BCL-2 inhibitors may represent a promising target approach considering the expression of BCL-2 family proteins in NB cells^5–8^, also confirmed by our small NB series. VEN addition can overcome NB cell resistance to anticancer drugs by restoring apoptosis^13^. Our experience confirms a variable BCL-2 expression in NB tissue that is independent from *BCL-2* genes status, suggesting a constitutive expression of BCL-2 in NB. Interestingly, some studies showed that the expression is higher in NBs with high-risk characteristics, namely Myc-N amplification and unfavorable histology, making VEN an attractive option especially for R/R NB^29,30^. Moreover, our data seems to suggest a possible better response in patients with diffuse BCL-2 expression considering the response in one patient with bone marrow clearance (Patient 1) and a relatively longer time to event. Intriguingly, the patients with higher BCL-2 expression experienced a longer periods of neutropenia. We can speculate and suppose that a diffuse BCL-2 expression could correlate to greater susceptibility to VEN, resulting in a more consistent response on the one hand and greater hematologic toxicity on the other hand.

Our data indicate that the continuous dosing of VEN with Cy-Topo is not tolerated due to cytopenia especially in heavily pre-treated patients. Despite this, a discontinuous dosing of VEN with at least two weeks of consecutive administration may be effective to achieve a response or stabilization of disease in R/R NB as observed in our series.

The SD response observed in this series of heavily pre-treated HR NB is encouraging. Further evaluation is needed to verify the role of VEN added to standard NB backbone in the R/R setting or as a bridge treatment to the innovative approaches. Evaluation of discontinuous dosing of VEN with Cy-Topo with at least two weeks of VEN consecutive administration in larger cohorts of heavily pre-treated patients is warranted. However, this therapeutic approach deserves to be tested in less pretreated patients that may tolerate a 28-day schedule of VEN administration as well. The degree of BCL-2 expression seems to correlate with response and is independent from the *BCL-2* genes status. This data needs to be confirmed in large series.

### Supplementary Information

Supplementary Information.

## Data Availability

The data that support the findings of this study are available from the corresponding author upon reasonable request.
