# Supplementary material for: Venetoclax plus cyclophosphamide and topotecan in heavily pre-treated relapsed metastatic neuroblastoma: a single center case series
Source: Sci Rep. 2023 Nov 7;13:19295. doi: 10.1038/s41598-023-44993-9 (PMC10630499; doi:10.1038/s41598-023-44993-9)

Venetoclax plus cyclophosphamide and topotecan in heavily pre-treated relapsed metastatic neuroblastoma: a single center case series

Maria Antonietta De Ioris[1 *], Francesco Fabozzi[1], Francesca Del Bufalo[1], Giada Del Baldo[1], Maria Felicia Villani[2], Maria Giuseppina Cefalo[1], Maria Carmen Garganese[2], Alessandra Stracuzzi[3], Federica Tangari[4], Arturo Maria Greco[4], Isabella Giovannoni[3], Roberto Carta[1], Maria Luisa D'Andrea[5], Angela Mastronuzzi[1], and Franco Locatelli[1,6]

[1] Department of Pediatric Hematology and Oncology and of Cell and Gene Therapy, Bambino Gesù Children's Hospital, IRCCS, Rome, Italy

[2] Nuclear Medicine Unit, Bambino Gesù Children's Hospital, IRCCS, Rome, Italy

[3] Pathology Unit, Bambino Gesù Children's Hospital, IRCCS, Rome, Italy

[4] Unit of Clinical Pharmacy, Bambino Gesù Children's Hospital, IRCCS, Rome, Italy

[5] Department of Imaging, Bambino Gesù Children's Hospital, IRCCS, Rome, Italy

[6] Department of Life Sciences and Public Health, Catholic University of the Sacred Heart

Supplementary Figure 1. MIBG scintigraphy pre-treatment (A, planar images and B, SPECT-CT images) and post treatment (C, planar images and D, SPECT-CT images) of Patient 1.

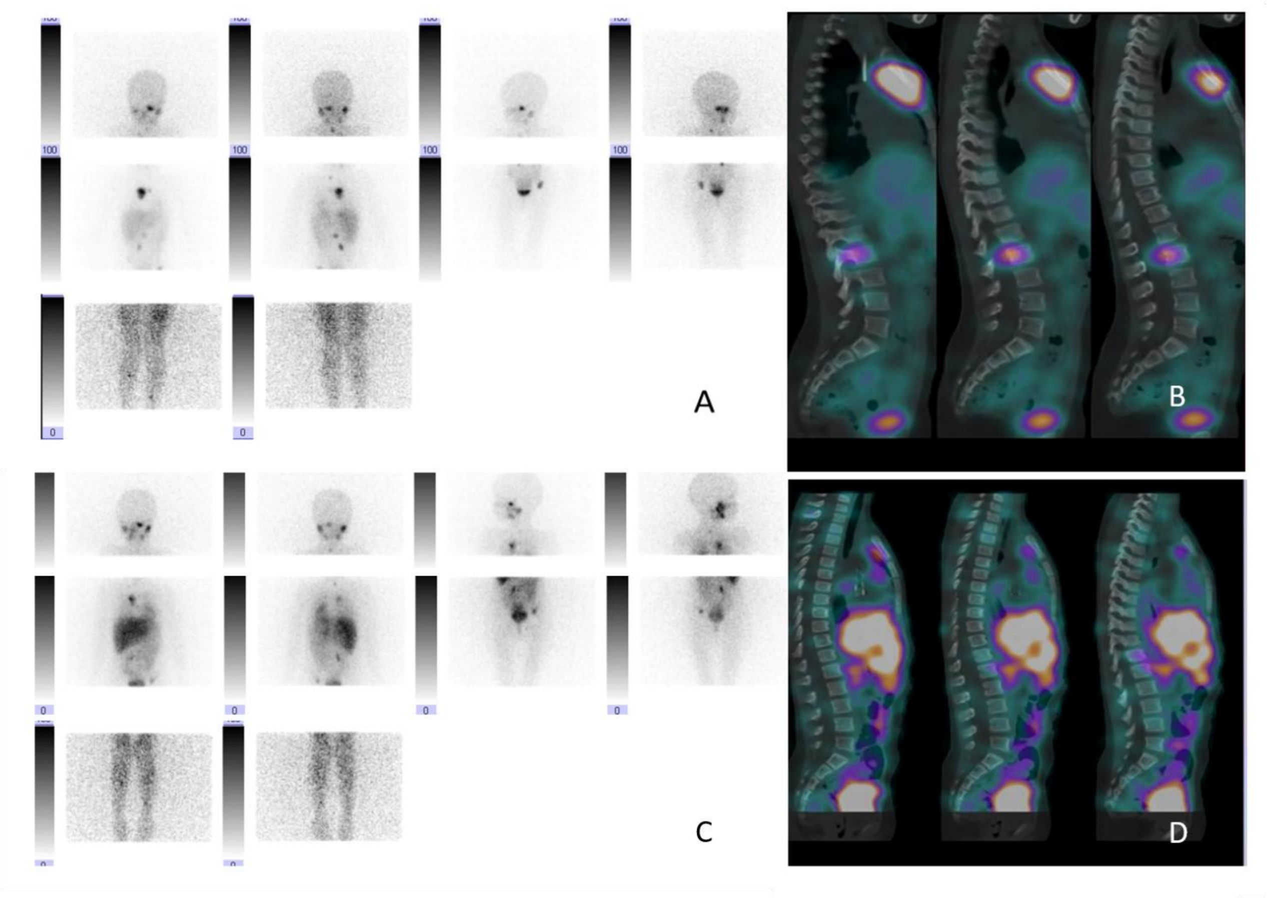

Supplementary Table 1. The spreadsheet table used to calculate the SIOPEN score.

	Skeleton Score	
	Pre treatment	Post treatment
Skull and facial bones		
Thoracic cage		
Right humerus		
Left humerus		
Right forearm		
Left forearm		
Spine		
Pelvis		
Right femur		
Left femur		
Right tibia/fibula		
Left tibia/fibula		
TOTAL		
SKELETAL SCORE<br><br>0 No abnormality<br>1 1focal lesion<br>2 2 focal lesions<br>3 3 focal lesions<br>4 Diffuse < 50% of bone or 3 focal lesions<br>5 Diffuse 50% — 95% of bone<br>6 Diffuse involving whole bone<br>X Unevaluable		

Supplementary Figure S2. BCL2 immunostain. Diffuse intense cytoplasmic staining is seen in patient 1 (A) and 2 (B), while mild to moderate staining is seen in patient 3 (C) and 4 (D)

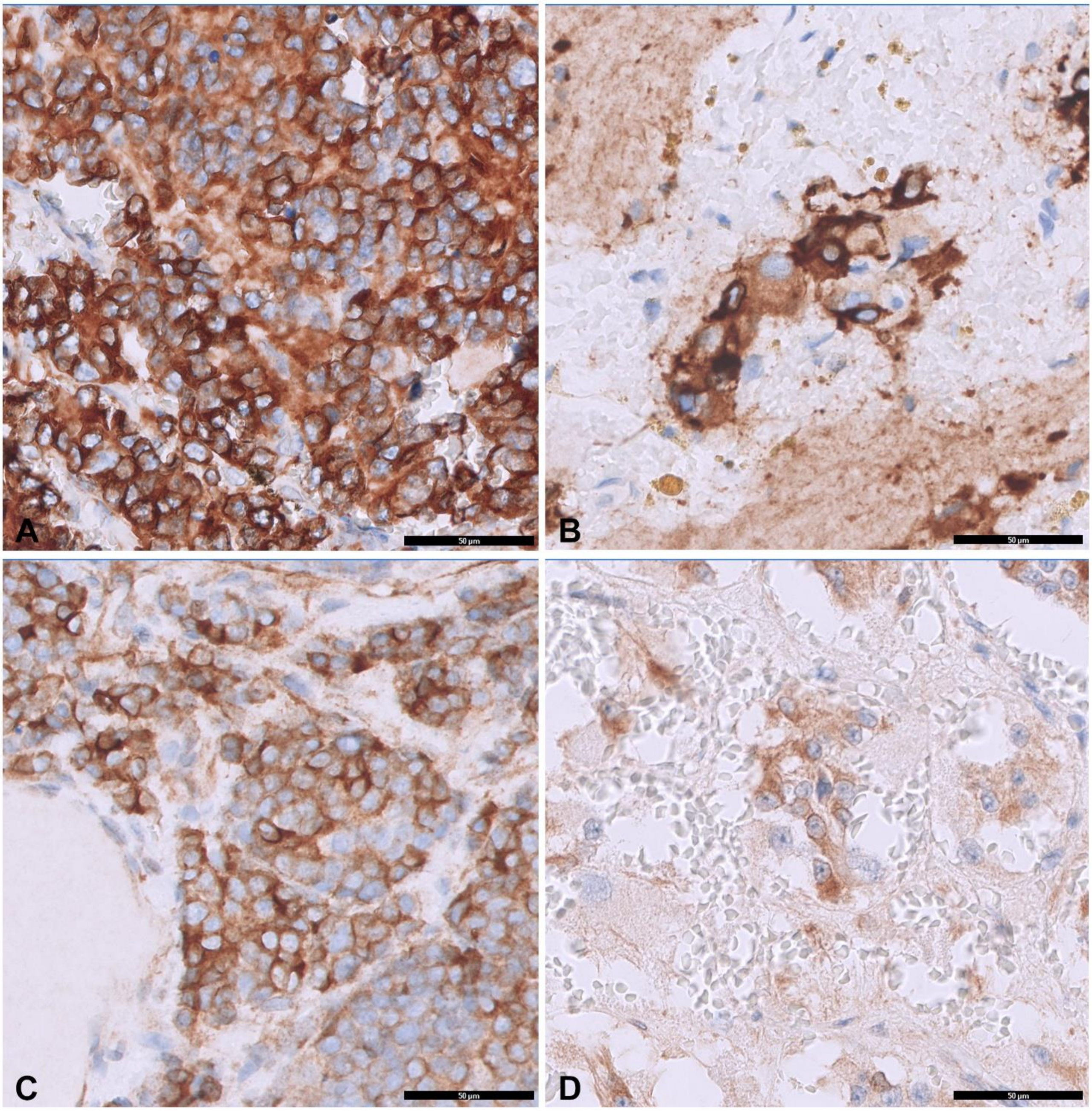

Supplement: Supplementary file 1 — Supplementary Information. [file 41598_2023_44993_MOESM1_ESM.pdf]
